# Risk factors for the emergence of multidrug-resistant bacteria in patients with head and neck infections: a retrospective analysis

**DOI:** 10.3389/froh.2026.1798339

**Published:** 2026-05-22

**Authors:** Dorottya Diana Kiss, Daniel Sandor Veres, Krisztina Marton, Zsolt Nemeth, Arpad Joob-Fancsaly, Katalin Kristof

**Affiliations:** 1 Department of Oro-Maxillofacial Surgery and Stomatology, Semmelweis University, Budapest, Hungary; 2 Department of Biophysics and Radiation Biology, Semmelweis University, Budapest, Hungary; 3 Department of Preclinical Dentistry, Semmelweis University, Budapest, Hungary; 4 Department of Laboratory Medicine, Semmelweis University, Budapest, Hungary

**Keywords:** antimicrobial resistance, head and neck infections, multidrug-resistant bacteria, odontogenic infections, oral cancer, risk factors, surgical site infections

## Abstract

**Background:**

The emergence and spread of multidrug-resistant (MDR) pathogens have been linked to excessive antibiotic use, as well as patient-related factors such as hospitalization. This study investigated the clinical determinants of antimicrobial resistance (AMR) and the occurrence of MDR bacteria.

**Methods:**

We evaluated 4,492 bacterial isolates from 1,719 patients and analyzed the association between resistance levels and various patient-related factors, including age, primary diagnosis, comorbidities, oral hygiene, smoking, alcohol use, and prior antibiotic therapy. AMR rate was graded from 0 to 3, and a mixed-effect continuation ratio was used for the analysis.

**Results:**

Patients with oral cancer (OR 1.38, 95% CI 1.06–1.80), a history of smoking (OR 1.19, 95% CI 1.01–1.41), immunosuppressive therapy (OR 1.36, 95% CI 1.06–1.75), insulin-treated diabetes (OR 1.57, 95% CI 1.05–2.35), or cardiovascular disease (OR 1.28, 95% CI 1.02–1.61) had significantly higher risk of infection with MDR bacteria. Prior antibiotic use (OR 1.44, 95% CI 1.24–1.68), older age (OR 1.01, 95% CI 1.00–1.01), and female sex (OR 1.28, 95% CI 1.09–1.49) were also significantly associated with progression to multidrug resistance.

**Conclusions:**

These findings suggest that, beyond selective pressure from antibiotics, individual patient characteristics may also contribute to the development of MDR bacteria in head and neck infections and. Clinicians should consider these factors during hospitalization, particularly regarding infection prevention and the responsible selection of antibiotics.

## Introduction

The emergence of multidrug-resistant (MDR) bacteria poses a major global health threat, particularly within the group referred to as ‘ESKAPE’ pathogens (*Enterococcus faecium, Staphylococcus aureus, Klebsiella pneumoniae, Acinetobacter baumannii, Pseudomonas aeruginosa*, and *Enterobacter* spp.) which are of critical concern in healthcare settings due to their diverse resistance mechanisms and widespread dissemination. Despite ongoing efforts, antibiotic development has failed to keep pace with the rapid evolution of resistant bacteria, as resistance frequently emerges shortly after the introduction of new drugs (1, 2).

Head and neck infections can lead to severe complications, and several predisposing factors, such as immunocompromised status, diabetes, age, systemic diseases, alcohol consumption, smoking, and poor oral health, have been associated with disease progression, prolonged hospitalization, or the need for intensive care (3–5). Certain findings suggest that conditions such as diabetes may even determine the causative pathogens of infections (6). Development of antimicrobial resistance (AMR), often resulting from prolonged use of antibiotics, can also contribute to extended hospitalization for patients with infections (7–9).

Studies have also investigated the possible risk factors for colonization with MDR pathogens in patients treated at long-term care facilities and intensive care units (10–12). ESKAPE pathogens are significant nosocomial agents and common causes of pneumonia, as well as urinary tract, intra-abdominal, and bloodstream infections (1). Although these pathogens have been extensively researched across various medical fields, only a limited number of studies focused on the prevalence of MDR pathogens in head and neck infections (13, 14).

Research in this area is particularly relevant as the oral cavity offers an optimal environment for biofilm formation, which not only promotes the persistence of bacteria but also facilitates the transmission of AMR genes. Intraoral biofilms may serve as reservoirs for MDR organisms, complicating the management of local infections and potentially contributing to the broader dissemination of AMR. Moreover, bacteremia can allow oral bacteria to enter the bloodstream, especially during surgical interventions, in patients with poor oral hygiene, or when chronic infections are present. Therefore, understanding and identifying the risk factors for MDR bacteria in head and neck infections is essential for selecting appropriate empirical antibiotic treatments, improving patient outcomes, reducing complications, and limiting the unnecessary use of broad-spectrum antibiotics (15–17)**.** In routine maxillofacial practice, clinicians encounter a broad spectrum of conditions—including infectious, oncologic, and therapy-related entities—often within the same clinical setting.

This study aimed to evaluate the prevalence of MDR bacteria and identify patient-related clinical risk factors contributing to their emergence in patients with head and neck infections, using a unified analytical approach across this patient cohort. To our knowledge, this represents the first large-scale study specifically examining risk factors for multidrug resistance in this population.

## Materials and methods

A retrospective analysis was conducted on patients treated for head and neck infections between 2018 and 2024 at the Department of Oro-maxillofacial Surgery and Stomatology, Semmelweis University (Budapest, Hungary). Sterile swabs were used to collect samples from various infection sites, including abscesses, surgical site infections (SSIs), and osteonecrosis sites, either intraorally or extraorally, depending on the location of the infection. The samples were delivered to the Department of Laboratory Medicine in transport medium (ESwab® 481C), where aerobic and anaerobic cultivation and antimicrobial susceptibility testing were performed. Bacterial identification was achieved using a matrix-assisted laser desorption ionization–time of flight mass spectrometry (MALDI-TOF MS) system (Bruker Ltd., USA) following cultivation. Results were evaluated according to the current EUCAST (European Committee on Antimicrobial Susceptibility Testing) guidelines (18).

### Data collection

Patient records were extracted from the electronic medical system of the university. The following factors were included in the medical history and relevant clinical data for analysis: age, sex, primary diagnosis, hospitalization during the treatment period, treatment-related factors (recurrence, foreign material—e.g., titanium plates, dental implants, bone graft material), cardiovascular disease (e.g., heart failure, ischemic heart disease, cerebrovascular disease, and arrhythmias), renal failure, prior antibiotic therapy, oral hygiene, immunosuppression, prior malignancy, chemotherapy, radiotherapy, diabetes (with or without insulin therapy), antiresorptive therapy, smoking (current tobacco use), chronic alcohol consumption, and high C-reactive protein (CRP) levels. Prior antibiotic therapy was defined as any systemic antibiotic use within the last 10 days before sampling, regardless of indication (e.g., therapeutic treatment of infection or perioperative prophylaxis). Prior malignancy referred to a history of cancer unrelated to the current primary diagnosis. Apart from chemotherapy, immunosuppression was defined as the use of immunosuppressive therapy, including biological therapies and corticosteroids. Antiresorptive therapy involved treatments such as bisphosphonates or denosumab. High CRP was defined as values exceeding 200 mg/L, a threshold commonly associated with severe bacterial infections and systemic inflammatory responses (19, 20). Patient confidentiality was ensured by assigning code numbers for data handling in accordance with Regulation 2016/679 of the European Parliament and of the Council on General Data Protection. No patients were excluded based on clinical or other criteria.

The resistance rate was classified from 0 to 3, based on the number of antimicrobial groups to which the bacteria were resistant. Multidrug resistance was defined as level 3 resistance, indicating resistance to ≥3 antibiotic classes. The antimicrobial groups examined included penicillins, carbapenems, cephalosporins, fluoroquinolones, macrolides, lincosamides (clindamycin), tetracyclines, glycopeptides, aminoglycosides, and trimethoprim/sulfamethoxazole. For statistical analysis, bacteria were classified as aerobic or anaerobic. As MDR strains were identified solely among aerobes, they were analyzed separately from anaerobes. Only bacterial genera with at least ten isolates per year were included in the final analysis; less frequently detected or clinically irrelevant genera were excluded. Recurrent isolates exhibiting identical resistance patterns within a one-year period in the same patient were also excluded from the analysis. Bacteria with similar characteristics were grouped to improve clarity. Statistical evaluation was conducted on twelve bacterial genera: *Actinomyces, Enterobacter* (including less frequent Enterobacterales e.g*., Citrobacter*, *Morganella*, *Proteus,* and *Serratia* spp.)*, Enterococcus, Escherichia, Fusobacterium, Haemophilus, Klebsiella, Prevotella, Pseudomonas, Staphylococcus, Streptococcus,* and *Veillonella*. *Staphylococcus* spp. were categorized into *S. aureus* and “other” staphylococci (primarily coagulase-negative staphylococci), while *Streptococcus* spp. were categorized into beta-hemolytic streptococci and “other” streptococci (primarily viridans group streptococci).

### Statistical analysis

As some patients had several samples taken and most of them had multiple bacterial species in their isolates, a mixed-effects continuation ratio (CR) model was employed for the ordinal outcome for aerobic bacteria, and a generalized linear mixed-effect model (glmm) was used for anaerobic bacteria to account for repeated measures from the same patients across multiple occasions. CR models estimate the odds of falling into a specific category vs. falling into that category or a higher one (21, 22). For clarity, results were reported in reciprocal form. Statistical analyses were conducted using R software (v4.5.0). Descriptive tables and plots were generated using the *table1* (v1.5.0) and *ggplot2* (v4.0.0) packages. The *mixed_model* function from the *GLMMadaptive* package (v0.9.7) was used, alongside the *glmmTMB* package (v1.1.12) for the glmm models. To display multiplicity-corrected comparison results for bacteria, the *emmeans* package (v1.11.2.8) was used. Potential two-way interactions were examined and considered for inclusion in the final model; however, none were found to be relevant or significant based on descriptive statistics, clinical relevance, and information criteria. The model estimated the probability of being in a specific resistance category conditional on having reached at least that category. This approach allowed us to evaluate how risk factors influenced the likelihood of advancing to higher resistance levels. Predicted probabilities were calculated for each threshold (Y ≥ 1, Y ≥ 2), and visualized using effect plots, stratified by key variables such as diabetes and prior antibiotic exposure, with 95% confidence intervals shown. While the CR model evaluates progression across all resistance levels, the findings are interpreted in terms of MDR due to its clinical relevance and role as the highest resistance category. Chemotherapy and radiotherapy showed high collinearity with variables related to malignancy (primary diagnosis of oral cancer and prior malignancy in medical history). To avoid multicollinearity in the main model, these treatment-related variables were excluded. A separate subgroup analysis was conducted including only patients with a current or past diagnosis of malignancy, to assess the association between chemotherapy, radiotherapy, and resistance. Additionally, stratified analyses were performed for the non-oncologic diagnostic groups (abscess and osteonecrosis) to explore potential differences in risk factors across clinically distinct entities, while oncologic patients were analyzed separately as described above.

For anaerobic bacteria, isolates with resistance level 2 were rare and no MDR bacteria were detected. Therefore, resistance was analyzed as a binary outcome, rather than using the ordinal resistance categorization applied to aerobic bacteria.

## Results

A total of 4,492 isolates from 1,719 patients were included in the final analysis. The primary diagnoses of the patients were categorized into three major groups: ‘abscess’ (dental origin), ‘necrosis’ and ‘tumor’. Most cases of necrosis were attributed to medication-related osteonecrosis or osteoradionecrosis; however, a few patients exhibited osteomyelitis without a known predisposing treatment history. The ‘tumor’ group included patients treated for oral cancer, primarily involving postoperative wound infections. The mean age of patients was 51.5 years, with a male-to-female ratio of 1.14. The distribution of resistance levels among isolates is summarized in Supplementary Table 1. The factors that were considered in the statistical model for evaluating the risk of MDR bacteria emergence are shown in Table 1.

**Table 1:** Factors considered in statistical model for MDR Bacteria risk assessment. .

Risk factors	n (%)
Primary diagnosis*	
Abscess	1,112 (64.5%)
Necrosis	419 (24.3%)
Tumor	193 (11.2%)
Hospitalization	904 (52.6%)
ICU treated (among hospitalized)**	23 (25.4%)
Cardiovascular disease	188 (10.9%)
Renal failure	31 (1.8%)
Diabetes	236 (13.7%)
Insulin therapy (among diabetics)	53 (22.5%)
Immunosuppression	165 (9.6%)
Prior malignancy	449 (26.1%)
Chemotherapy	287 (16.7%)
Radiotherapy	278 (16.2%)
Antiresorptive therapy	298 (17.3%)
Smoking	547 (31.8%)
Chronic alcohol consumption	138 (8.0%)
Recurrence of disease	403 (23.4%)
Foreign material presence	147 (8.5%)
Prior antibiotic therapy (per sample)***	60.3%
High CRP (>200)	96 (5.6%)
Poor oral hygiene	283 (16.5%)

*Five patients were treated for both necrosis and tumor as separate diagnoses at different time periods.

**Although patients treated in the Intensive Care Unit (ICU) are highlighted in this table, ICU treatment was not included in the analysis due to the small number of cases. Of the 23 patients treated in the ICU, 5 developed sepsis.

***Prior antibiotic therapy was assessed based on 2,378 samples taken during the study period. The percentage reflects the proportion of samples where patients received previous antibiotic treatment, not the individual patients.

### Resistance patterns and associated risk factors among aerobic bacterial isolates

**Figure 1:**
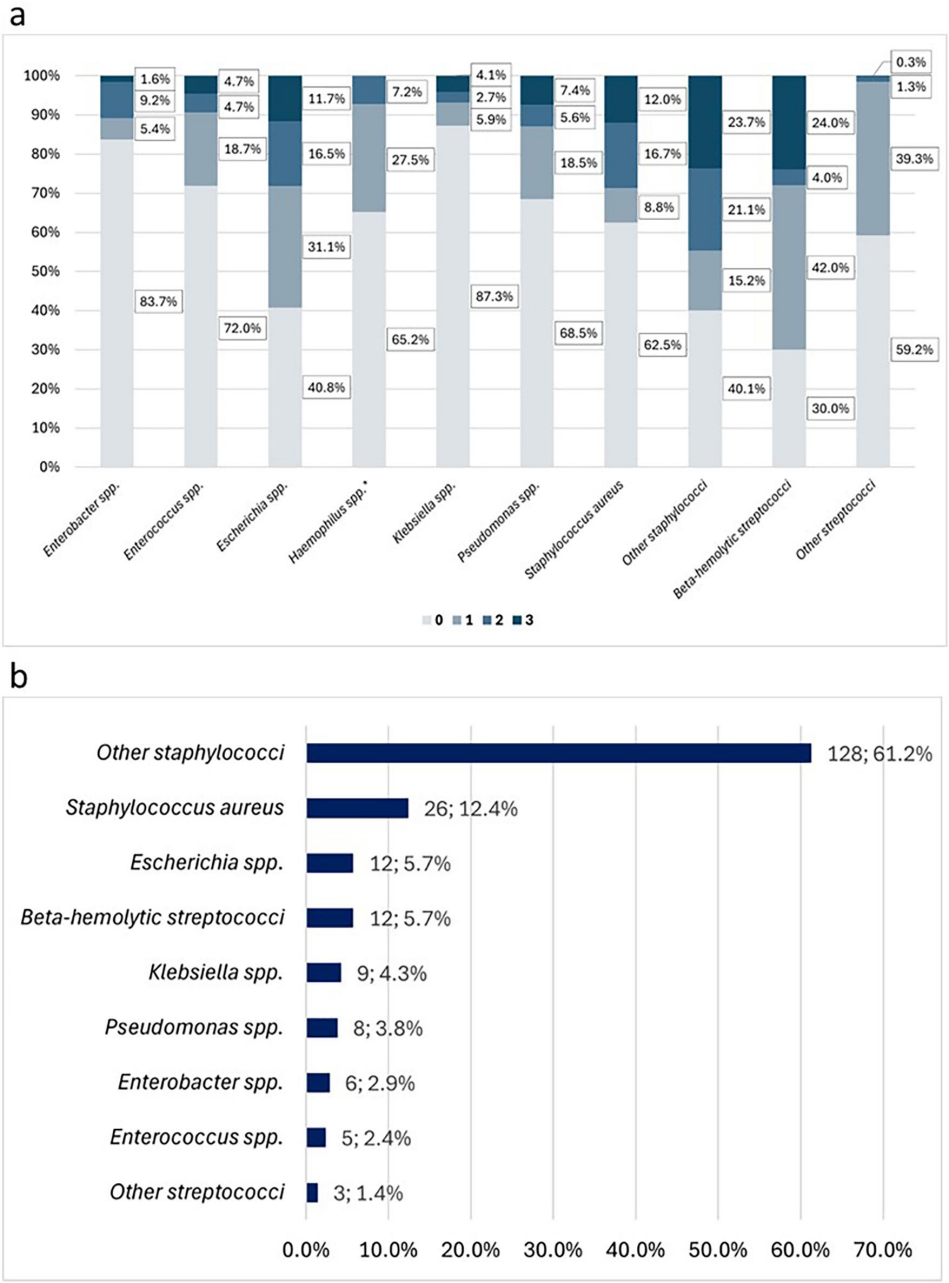
AMR among aerobic bacteria. “other staphylococci” primarily includes coagulase-negative staphylococci, while “other streptococci” mainly consists of viridans group streptococci. **(a)** Proportions of resistance levels (0-3) among aerobic bacterial isolates. **(b)** Distribution of species among MDR bacteria only. *No MDR bacteria (level 3) were isolated.

Among aerobic bacteria, all species harbored MDR pathogens, except for *Haemophilus* spp. The highest ratio was observed in beta-hemolytic streptococci (24.0%) and coagulase-negative staphylococci (23.7%). ESKAPE pathogens showed slightly lower percentages of MDR bacteria, with *S. aureus* and *Pseudomonas* spp. exhibiting the highest rates among them (12.0% and 7.4%, respectively). Overall resistance rates (categories 1–3) were highest in beta-hemolytic streptococci, coagulase-negative staphylococci, and *Escherichia* spp. (Figure 1). The distribution of bacterial species among MDR strains across the primary diagnoses is shown in Supplementary Figures 1, 2. Antimicrobial susceptibility patterns are presented in Supplementary Table 2.

Regarding aerobic bacteria, older age, female sex, tumor as the primary diagnosis, immunosuppression, diabetes requiring insulin therapy, cardiovascular disease, smoking, and prior antibiotic therapy were significantly associated with increased odds of progressing to higher AMR categories in the CR model (Table 2, Supplementary Figure 3). The risk of having MDR bacteria was significantly higher in coagulase-negative staphylococci and beta-hemolytic streptococci compared to other species (Supplementary Figure 4). In the subgroup analysis of patients with malignancies, neither chemotherapy nor radiotherapy was significantly associated with progression to higher resistance levels (Supplementary Table 3). Additional stratified analyses by primary diagnostic group are provided in Supplementary Tables 4, 5. Notably, in the subgroup analysis of abscess patients, the presence of foreign material emerged as a significant risk factor, highlighting its importance in infections involving biofilm formation on foreign materials such as osteosynthesis plates or implant surfaces. Across these subgroup models, the direction of the observed effects remained largely consistent with the overall analysis, although fewer variables reached statistical significance, probably due to the reduced sample size.

**Table 2:** Association of variables with the odds of progression to higher AMR categories in the CR model.

Variable	OR	95% CI	*p*-value
Primary diagnosis			
Abscess (ref)	—	—	
Necrosis	1.11	0.84–1.48	0.46
Tumor	1.38	1.06–1.80	**0**.**017**
Sex			
Male (ref)	—	—	
Female	1.28	1.09–1.49	**0**.**002**
Hospitalization	1.08	0.90–1.29	0.41
Recurrence	1.14	0.97–1.36	0.12
Foreign material	1.23	0.97–1.56	0.086
Prior antibiotics	1.44	1.24–1.68	**<0**.**001**
Prior malignancy	1.13	0.88–1.44	0.34
Renal failure	0.92	0.51–1.67	0.79
Poor oral hygiene	1.11	0.90–1.36	0.33
Diabetes			
Non-diabetic (ref)	—	—	
Diabetic without insulin	1.03	0.81–1.30	0.81
Diabetic with insulin	1.57	1.05–2.35	**0**.**029**
Immunosuppression	1.36	1.06–1.75	**0**.**016**
Smoking	1.19	1.01–1.41	**0**.**038**
Alcohol	0.89	0.68–1.17	0.42
High CRP	1.16	0.84–1.61	0.36
Cardiovascular disease	1.28	1.02–1.61	**0**.**036**
Antiresorptive therapy	0.81	0.62–1.05	0.12
Age	1.01	1.00–1.01	**0**.**028**

Shown are odds ratios (OR), 95% confidence intervals (CI), and *p*-values. For categorical variables, one category was used as the reference (ref), but does not have an associated OR, CI, or *p*-value in the table. Results for chemotherapy and radiotherapy, analyzed in a separate model including only patients with malignancy, are presented in the Supplementary Material (Supplementary Table 2).

Statistically significant *p*-values are shown in bold.

**Figure 2:**
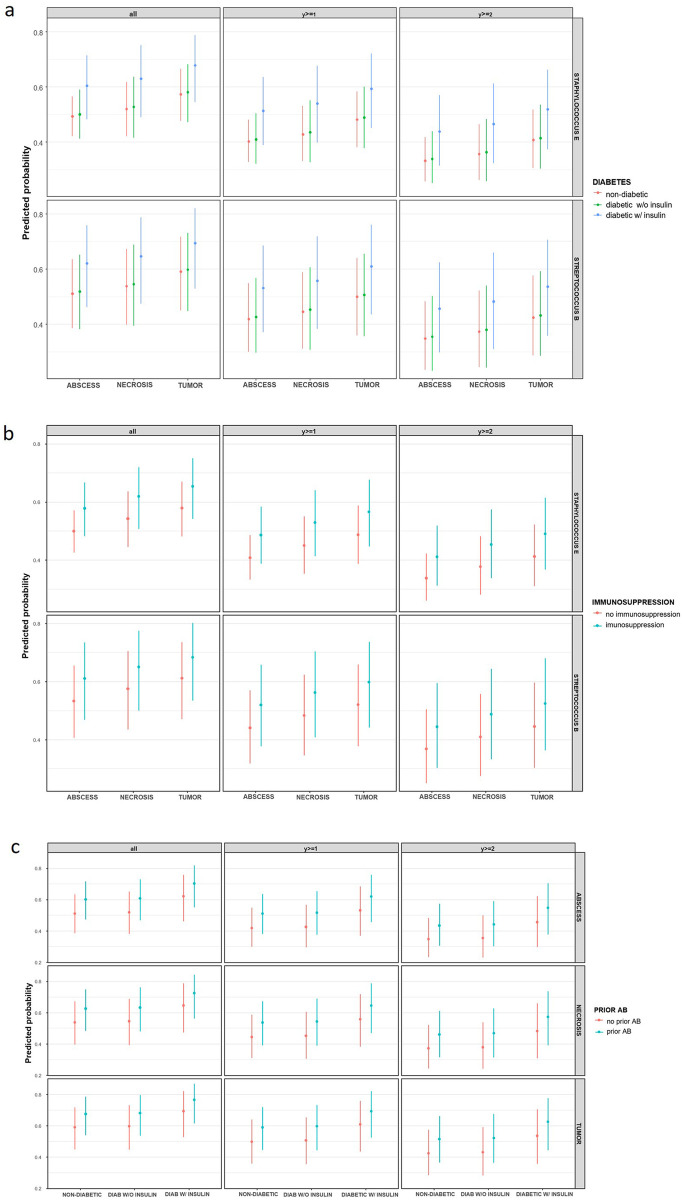
Predicted probabilities of increasing AMR levels across clinical risk factors. CR models were used to estimate the predicted probability of bacterial resistance across increasing resistance thresholds: overall resistance (all), y ≥ 1, and y ≥ 2. **(a)** Predicted probabilities by primary diagnosis (abscess, necrosis, tumor) and diabetes status (non-diabetic, diabetes w/o insulin, diabetes w/ insulin). **(b)** Predicted probabilities by primary diagnosis and immunosuppression status. **(c)** Combined effects of primary diagnosis, diabetes status, and prior antibiotic exposure. Across all panels, the highest predicted probabilities were consistently seen in patients with oral cancer, insulin-requiring diabetes, and prior antibiotic use — approaching ∼0.8 in the overall resistance group and remaining above 0.6 for higher resistance levels. Beta-hemolytic streptococci showed slightly higher probabilities than coagulase-negative staphylococci across similar conditions. STREPTOCOCCUS B: beta-hemolytic streptococci, STAPHYLOCOCCUS E: coagulase-negative staphylococci (“other”).

The effect plots showed that patients diagnosed with oral cancer had higher predicted probabilities of resistance across all thresholds compared to those with abscess or necrosis. Factors such as diabetes requiring insulin, immunosuppression, and prior antibiotic exposure were all associated with increased likelihood of higher resistance levels. Beta-hemolytic streptococci exhibited slightly higher resistance probabilities than coagulase-negative staphylococci. The highest resistance probabilities were observed in patients presenting simultaneously with a tumor diagnosis, diabetes requiring insulin, and prior antibiotic exposure (Figure 2).

### Assessment of anaerobic bacterial isolates and related risk factors

**Figure 3:**
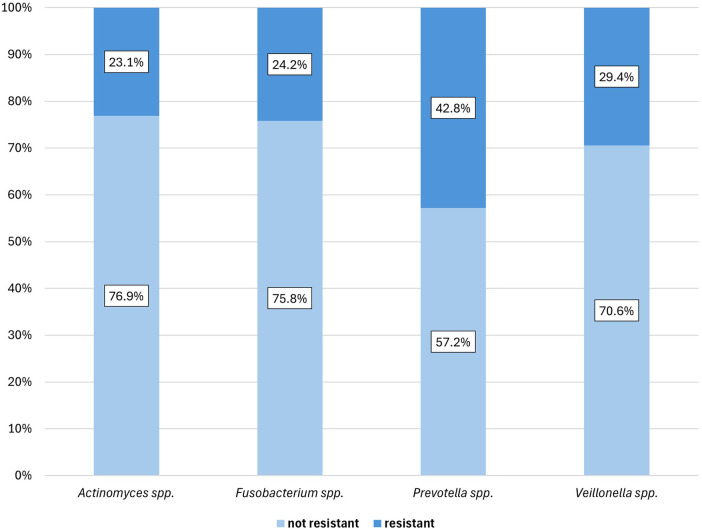
AMR among anaerobic bacterial isolates.

Although resistance levels up to 2 were observed among anaerobic bacteria, resistance was analyzed as a binary outcome (resistant vs. not resistant). *Prevotella* spp. showed significantly higher resistance rates compared to other species, while *Actinomyces* spp. exhibited resistance to at most one antimicrobial class. Among clinical factors, only prior antibiotic therapy and renal failure were significantly associated with resistance (Figure 3, Supplementary Table 6 and Supplementary Figure 5).

### Observations on inadequate antibiotic use and allergies

Inadequate antibiotic therapy was relatively rare, observed in only 48 patients (2.8%), of whom only one had an associated MDR isolate. However, most cases involved the misuse of clindamycin (*n* = 46; 95.8%). Among patients who received clindamycin, 19.2% were prescribed inappropriate doses, and 70.3% had no documented penicillin allergy, indicating that it was frequently used as a first-line agent. Self-reported allergies to antimicrobial agents were observed in 207 patients (12.0%), with penicillin allergy being the most common (*n* = 151; 72.9%). Other reported allergies included clindamycin, cephalosporins, trimethoprim/sulfamethoxazole, fluoroquinolones, tetracyclines, and macrolides. Allergy to multiple antimicrobial agents was documented in 22 cases. Seven patients developed *Clostridium difficile* infection during the treatment period; prior antibiotics associated with these cases included carbapenems, fluoroquinolones, amoxicillin or clindamycin and in one case, linezolid.

### Progression to multidrug resistance and occurrence of multiple MDR isolates in individual patients

Additionally, we identified a subset of cases (*n* = 20) in which the same bacterial species was repeatedly isolated from a patient during the treatment period, with resistance progressing to level 3 (MDR). Due to this limited sample size, no statistical analysis was performed; however, a descriptive summary of clinical and treatment-related factors is provided in Table 3. Among the 20 cases, patients were predominantly older; hospitalization and prior antibiotic exposure were frequent; most isolates were coagulase-negative staphylococci with resistance progressing to level 3 within 90 days; comorbidities and prior malignancy were common.

**Table 3:** Patients from whom the same bacterial species was repeatedly isolated, showing progression to resistance level 3 (MDR) during the treatment period. .

Patient ID	Pathogen	Progr	Days	Sex	Age	Primary Dg	Hospit	Recur	Prior AB	Type of AB				Prior malignancy	CVD	Poor oral hygiene	Chemo	Radio	Diabetes	Immun	Smoking	CRP high	Antires
										AMX	CL	FQ	Other										
1	CoNS	0 to 3	31-90	M	86	NECR	+	+	+	+	-	-	-	+	+	-	-	-	+	-	-	-	+
2	EB	2 to 3	0–30	F	70	TU	+	+	+	+	-	-	-	+	-	+	-	+	+	-	+	-	-
3	E. coli	1 to 3	31–90	F	78	NECR	+	+	+	+	-	+	-	+	+	-	-	+	-	-	+	-	-
4	PM	1 to 3	0–30	M	36	ABSC	++	+	+	-	-	-	+	-	-	-	-	-	-	-	+	+	-
5	CoNS	2 to 3	31–90	M	72	TU	+	+	+	-	-	-	+	-	-	-	+	+	-	-	+	-	-
6	CoNS	0 to 3	31–90	F	79	NECR	+	+	+	+	-	-	-	+	-	-	+	+	-	+	-	-	-
7	EB	0 to 3	0–30	F	72	TU	++	-	+	-	-	+	-	+	+	-	+	-	-	+	+	-	-
8	CoNS	2 to 3	0–30	M	30	TU	+	+	+	+	-	-	-	-	-	-	+	+	-	+	-	-	-
9	CoNS	0 to 3	>90	M	56	NECR	+	-	+	+	-	-	-	+	-	-	+	-	-	-	-	-	+
10	PM	0 to 3	0–30	M	69	TU	+	+	+	+	-	-	+	-	+	-	-	-	+	-	+	-	-
11	CoNS	1 to 3	31–90	M	66	ABSC	+	-	-	-	-	-	-	-	-	-	-	-	-	-	-	-	-
12	CoNS	2 to 3	31–90	M	64	NECR	+	-	+	-	+	-	-	+	-	-	-	+	-	-	+	-	-
13	CoNS	0 to 3	0–30	M	63	ABSC	+	-	+	+	+	-	-	-	-	-	-	-	-	-	-	+	-
14	CoNS	0 to 3	0–30	F	28	ABSC	+	+	+	-	-	+	-	-	-	+	-	-	-	-	+	+	-
15	CoNS	0 to 3	0–30	M	20	ABSC	++	+	+	-	-	-	+	-	-	+	-	-	-	-	-	+	-
16	EB	0 to 3	0–30	F	65	ABSC	++	+	+	-	-	+	+	-	-	-	-	-	+	-	-	+	-
17	E. coli	2 to 3	31–90	M	68	NECR	+	-	+	+	-	-	-	+	+	-	+	-	-	-	-	-	+
18	CoNS	2 to 3	>90	M	69	NECR	+	+	+	+	-	-	+	+	-	-	-	-	+	+	-	-	+
19	CoNS	0 to 3	31–90	F	75	NECR	+	+	-	-	-	-	-	+	+	-	+	+	+	-	+	-	-
20	CoNS	1 to 3	>90	F	79	NECR	+	+	+	+	-	-	-	+	-	-	+	+	-	-	-	-	+
	CoNS: 65.0%	0: 50.0% 1: 20.0% 2: 30.0%	<90: 85.0%	M:F 1.5	Mdn 69	A: 30.0% N: 45.0% T: 25.0%	100.0%	70.0%	90.0%	50.0%	10.0%	20.0%	30.0%	55.0%	30.0%	15.0%	40.0%	40.0%	30.0%	20.0%	45.0%	25.0%	25.0%

Patients are numbered sequentially for clarity; these do not correspond to original dataset identifiers. Bacteria isolated in these cases included coagulase-negative staphylococci (CoNS), *Enterobacter* spp. (EB), *Escherichia coli* (*E. coli*), and *Pseudomonas* spp. (PM). The relevant factors included in this descriptive analysis were: progression to level 3 resistance (Progr), time interval between sampling grouped into 0–30, 31–90 and over 90 days (Days), sex, age, hospitalization during treatment period (Hospit, ++ meaning treatment in ICU), primary diagnosis (Primary Dg) – abscess (ABSC), necrosis (NECR), tumor (TU), recurrence (Recur), previous antimicrobial therapy within 10 days prior to the isolation of MDR bacteria (Prior AB) with the type of antimicrobials (Type of AB): amoxicillin (AMX), clindamycin (CL), fluoroquinolones (FL), Other (e.g.: macrolide, metronidazole, trimethoprim/sulfamethoxazole) and selected clinical factors including prior malignancy, cardiovascular disease (CVD), poor oral hygiene, chemotherapy (Chemo), radiotherapy (Radio), diabetes, immunosuppression (Immun), smoking, high CRP and antiresorptive therapy (Antires). Variables with very low prevalence in this subgroup, such as chronic alcohol consumption or foreign material presence, were not included in the table.

Another subset of patients (*n* = 18) and their clinical characteristics are summarized in Table 4. In this cohort, most patients were hospitalized and frequently pretreated with antibiotics, commonly presenting with oral cancer as the primary diagnosis; smoking and comorbidities were also frequent.

**Table 4:** Patients from whom several MDR bacteria were isolated during the treatment period.

Patient ID	Pathogen	Sex	Age	Primary diagnosis	Clinical factors	Prior antibiotic therapy
1	SA, CoNS	F	72	NECR	Hospitalization, Foreign material, Smoking, Antiresorptive therapy	amoxicillin+metronidazole
2	EB, EC, E. coli, SA	F	70	TU	Hospitalization, Prior malignancy, Poor oral hygiene, Radiotherapy, Diabetes, Smoking	amoxicillin
3	SA	M	55	NECR	Foreign material, Chronic alcohol consumption	amoxicillin+metronidazole
	CoNS					No
4	PM, CoNS	M	36	ABSC	Hospitalization**, Smoking	piperacillin/tazobactam
5	E. coli	M	64	TU	Hospitalization, Diabetes, Smoking	amoxicillin + metronidazole
	CoNS					imipenem
6	PM, CoNS	F	72	TU	Hospitalization**, Prior malignancy, Cardiovascular disease, Chemotherapy, Immunosuppression, Smoking	clindamycin, cephalosporin
	EB					fluoroquinolone
7	*KL	F	61	TU	Hospitalization, Smoking, Antiresorptive therapy	No
	SA					amoxicillin
8	SA, CoNS, EC	F	75	TU	Hospitalization, Foreign material, Radiotherapy, Diabetes	trimethoprim/sulfamethoxazole
9	E coli, CoNS	M	77	NECR	Hospitalization, Foreign material, Prior malignancy, Radiotherapy, Immunosuppression, Antiresorptive therapy	amoxicillin
10	KL, CoNS	F	57	ABSC	Prior malignancy, Chemotherapy, Immunosuppression, Antiresorptive therapy	piperacillin/tazobactam
11	E. coli, KL, SA	M	65	ABSC	Cardiovascular disease	No
12	BHS	F	30	NECR	Hospitalization, Smoking	amoxicillin
	CoNS					No
13	PM, CoNS	M	56	ABSC	Hospitalization**, Poor oral hygiene, Smoking, Chronic alcohol consumption	piperacillin/tazobactam
14	PM	M	83	TU	Hospitalization**, Prior malignancy, Cardiovascular disease, Radiotherapy	cephalosporin
	EC					No
15	E. coli, CoNS	M	65	NECR	Hospitalization, Prior malignancy, Chemotherapy, Radiotherapy, Smoking	No
16	CoNS	F	65	ABSC	Hospitalization**, Diabetes	amoxicillin + metronidazole
	EB					rifampicin,fluoroquinolone
17	CoNS, SA	F	82	TU	Hospitalization**, Cardiovascular disease, Diabetes	amoxicillin
	PM					glycopeptide, carbapenem
18	EB	M	74	TU	Hospitalization, Poor oral hygiene, Diabetes, Smoking	amoxicillin
	CoNS					No

This table includes selected clinical factors considered relevant to the presence of multiple MDR bacterial isolates. Patients are numbered sequentially for clarity; these do not correspond to original dataset identifiers. Bacteria isolated in these cases included *S. aureus* (SA), coagulase-negative staphylococci (CoNS), *Enterobacter* spp. (EB), *Enterococcus* spp. (EC), *Escherichia coli* (*E. coli*), *Pseudomonas* spp. (PM), *Klebsiella* spp. (KL), beta-hemolytic streptococci (BHS). Primary diagnoses were: abscess (ABSC), necrosis (NECR), tumor (TU). Where prior antibiotic therapy is listed in separate rows for the same patient, it corresponds to the sequence of isolates in the respective rows. For simplicity, ‘amoxicillin’ refers to amoxicillin/clavulanic acid.

*This patient had a MDR *Klebsiella pneumoniae* isolate that was detected again approximately one year after the initial finding, indicating persistent colonization or reinfection with a MDR strain.

**ICU-treated patients, one patient (14) had sepsis.

There were some overlaps between these two patient subsets, with four patients appearing in both groups. These patients had recurring isolates of the same species that developed multidrug resistance over time, as well as simultaneous colonization or infection with multiple MDR species.

## Discussion

This retrospective analysis evaluated the prevalence of MDR pathogens in head and neck infections, as well as the potential risk factors associated with their emergence. Coagulase-negative staphylococci and beta-hemolytic streptococci showed the highest percentages of MDR pathogens (level 3) across all aerobic bacteria studied. Among the ESKAPE pathogens, *S. aureus* and *Pseudomonas* spp. isolates exhibited the highest proportion of MDR bacteria. The number of *A. baumannii* isolates fell below the threshold for statistically valid analysis and was therefore not included. However, it is noteworthy that one patient developed a multidrug-resistant *A. baumannii* infection during the study period, which ultimately resulted in a fatal outcome. No MDR strains were identified in *Haemophilus* spp. or among anaerobic bacteria; however, *Prevotella* spp. displayed significantly higher resistance compared to other anaerobic species.

Our study revealed several possible risk factors that could be associated with the emergence of MDR pathogens, including older age, female sex, tumor as the primary diagnosis, immunosuppression, diabetes requiring insulin therapy, cardiovascular disease, smoking, and prior antibiotic therapy. Although age exhibited a statistically significant association with the outcome in this cohort, the effect size was small and may have limited clinical relevance.

The effect plots further illustrated that combinations of key risk factors, such as diabetes requiring insulin therapy, prior antibiotic exposure, and tumor as a primary diagnosis, were associated with notably higher predicted probabilities of resistance.

While infection with MDR bacteria can be associated with inadequate antibiotic therapy (23), our study found instances of inappropriate dosing in only 48 patients, with only one demonstrating an associated MDR isolate. Due to the limited number of such cases, antibiotic misuse was not included as a risk factor in the main statistical analysis. However, the inappropriate use of clindamycin could contribute to the rising resistance observed in head and neck infections, possibly leading to treatment failure (24). Notably, perioperative clindamycin use has also been recognized as a risk factor for SSI in this region (25).

In addition to the main analysis, we identified 20 patients with repeated isolation of the same bacterial species during the treatment period at our clinic, with documented progression to level 3 resistance (MDR). While the limited number of cases only allowed for descriptive analysis, it provided insights into clinical characteristics associated with the development of resistance. Sampling intervals varied between patients depending on recurrence, clinical status, and follow-up schedules, thus resistance progression could only be estimated based on the duration between isolate collections. Nearly half of the patients showed progression to level 3 resistance over a relatively short time frame (0–30 days), suggesting a potential pattern of rapid resistance emergence during the treatment period. Hospitalization and prior antibiotic exposure appeared as primary contributing factors, present in the majority of cases. While antibiotic use can promote rapid bacterial adaptation through selective pressure, resistance progression may also result from the acquisition of already resistant strains within the hospital environment (26–28). Additionally, approximately half of the patients had prior malignancies or were smokers, suggesting that these factors may also play a role in resistance development.

In another subset of 18 patients with multiple MDR isolates, hospitalization and prior antibiotic treatment were also frequently observed, followed by smoking, prior malignancies, and diabetes. Although male sex has occasionally been reported as a potential risk factor for MDR bacterial infections (29, 30), our findings revealed a significantly higher risk among female patients.

Hospitalization and prior antibiotic use have been consistently identified as risk factors for MDR bacterial infections in numerous studies, particularly in cases involving ESKAPE pathogens. Older age is also frequently associated with these infections, but the findings are not consistent across studies (10, 12, 23, 29, 30). This variability may be explained by immunosenescence and a greater burden of comorbidities, both of which can increase susceptibility to infection and the risk of acquiring resistant pathogens (31). Research conducted by Mody et al. (32) highlighted potential sources of MDR bacterial transmission in nursing homes, including the hands of both residents and healthcare professionals, as well as contaminated environmental surfaces, with transmissions likely occurring during interactive visits.

In addition to prior hospitalization and antibiotic exposure, Shelke et al. (33) found that cancer patients undergoing chemotherapy or surgery were more likely to have MDR bacterial infections. As a primary diagnosis, oral cancer (‘tumor’ group) was identified as a significant predictor for the presence of MDR bacteria. This might be attributed to a combination of several contributing factors, including immunosuppression, prior oncological treatments, repeated hospitalizations, malnutrition and tissue disruption, that can collectively increase the risk of having MDR bacteria (34). Although patients with osteonecrosis (‘necrosis’ group) often share similar risk factors such as a history of malignancy, hospitalization, or prior antibiotic use, this condition was not found to be a significant predictor in our analysis. This suggests that the tumor site and its postoperative environment are associated with greater susceptibility to MDR bacterial colonization or infection, potentially due to alterations in the local microbiome, although the exact source of these bacteria remains unclear (35). Jiang et al. (36) found that cancer patients had considerably high risk of nosocomial infections caused by MDR bacteria. They also found smoking to be significantly associated with the presence of MDR bacteria, aligning with our results, although it is not consistently recognized as a risk factor in the broader literature. Given the known immunomodulatory effects of smoking and its impact on mucosal defenses, these findings support the need to further explore the potential contribution of smoking to MDR bacterial colonization. Notably, their analysis also found that radiotherapy was not significantly associated with MDR infections, which is consistent with our findings.

Despite hospitalization not showing a significant association with MDR infections in the overall analysis, in two specific patient subgroups—those with the same species progressing to MDR and those with multiple MDR pathogens—hospitalization was documented in the majority of cases, with several requiring ICU care. This suggests that in more severe clinical scenarios, hospitalization may remain an important contributing factor.

Cardiovascular disease is not commonly highlighted as a risk factor for MDR infections in the literature, possibly due to differences in the definition of comorbidities; and when referenced, the types of cardiovascular diseases are often not clearly specified (37, 38). In our analysis, we applied a more specific definition, limited to ischemic heart disease, cerebrovascular disease, heart failure, and arrhythmias, and found a significant association with the presence of MDR bacteria. These findings suggest that certain cardiovascular conditions may play a more direct role in susceptibility and should be further explored as distinct risk factors.

2Foreign materials such as titanium plates, dental implants or bone grafts were also evaluated as potential risk factors for MDR bacterial infections. While the association did not reach statistical significance in the main analysis, the observed trend aligns with the recognized role of biofilm formation in promoting AMR, which was further supported by the subgroup analysis of abscess patients, where foreign material emerged as a significant risk factor. Previous studies in other clinical settings have primarily focused on traditional indwelling medical devices (e.g., catheters, tracheostomy tubes), where surfaces supporting biofilm formation are established risk factors for MDR infections (10, 30, 36, 39). Our findings suggest that foreign materials within the oral cavity may similarly serve as niches for resistance development and colonization by MDR bacteria. This association is particularly noteworthy given that *Staphylococcus epidermidis* and other coagulase-negative staphylococci, known for their biofilm-forming capabilities, were the most frequently isolated MDR bacteria. The role of *S. epidermidis* in oral biofilms as a reservoir for resistance genes has been documented in previous studies (24, 40), further supporting the hypothesis that intraoral foreign materials can facilitate the emergence of AMR.

Several studies have investigated the potential risk factors contributing to the severity and complications of head and neck infections. Although these studies did not specifically focus on MDR bacteria, they identified risk factors such as older age, the presence of underlying diseases—particularly diabetes—as being associated with more severe outcomes. One study also found an association between poor oral hygiene and more severe outcomes (3–5). In our investigation, it was not found to be a significant risk factor for the presence of MDR bacteria. This discrepancy may be partly explained by our stringent definition, as only patients with markedly neglected oral hygiene were categorized as such. A literature review of ESKAPE pathogens in head and neck infections found that *S. aureus* was the most commonly detected bacteria among ESKAPE pathogens and patients with immunosuppression, including diabetes, were more susceptible to infections and complications from this group of pathogens (41). Hamill et al. (13) identified MRSA as the most frequent pathogen in their study of SSIs following free flap reconstruction surgeries in oral cancer patients. They reported that neither the type nor the duration of perioperative antibiotic prophylaxis was associated with the development of postoperative infections or MDR organisms.

While immunosuppression has been recognized as a risk factor for MDR infections (23, 29, 42), its precise definition remains inconsistent in the literature. The present study distinguished between patients receiving immunosuppressive medications (e.g., corticosteroids or biological agents) and those with other conditions that may lead to immunosuppression, such as chemotherapy, radiotherapy, or diabetes. Adopting this narrower definition, immunosuppression was found to be a significant risk factor for MDR infections; however, neither chemotherapy nor radiotherapy showed a significant association with MDR bacteria. Nonetheless, radiotherapy has been identified as a risk factor for SSIs in the head and neck region (25). Insulin-treated diabetes was also significantly associated with MDR infections, consistent with some prior reports (30, 43, 44), though other studies did not observe this association (12, 23, 29, 36, 45). Notably, in our study, we distinguished between diabetic patients who required insulin therapy and those who did not, demonstrating that only patients on insulin therapy were at a significantly higher risk of carrying MDR bacteria.

In addition to the commonly reported risk factors such as older age, immunosuppression, prior antibiotic use, and diabetes, our study identified female sex, oral cancer as the primary diagnosis, smoking, and cardiovascular disease as significant risk factors for the emergence of MDR bacteria. Notably, among patients with diabetes, only those requiring insulin therapy exhibited significantly higher risk. In our analysis, immunosuppression was defined as the use of immunosuppressive medications, such as corticosteroids or biological agents, excluding chemotherapy, as well as underlying conditions like HIV or autoimmune diseases. While previously reported factors like chemotherapy and hospitalization were not statistically significant in our overall cohort, findings from two subset analyses suggest that these factors, particularly hospitalization, may still play an important role in more severe cases.

In managing head and neck infections, clinicians should consider relevant comorbidities and prior antibiotic exposure to better address the risk of MDR bacteria. Patients presenting with severe infections, particularly those requiring hospitalization, may benefit from early microbiological screening and, where appropriate, rapid molecular testing to guide timely adjustment of antimicrobial therapy. Incorporating these risk profiles into empirical treatment decisions could help reduce the inappropriate or excessive use of broad-spectrum antibiotics, a key contributor to the development and spread of MDR bacteria. In addition, implementing infection prevention measures, such as improved hygiene practices, environmental cleaning, and strict adherence to isolation precautions, as recommended in hospital settings, can help reduce the transmission of multidrug-resistant bacteria (28). Given the diversity of conditions encountered in this field, these findings may support clinical decision-making across different patient groups.

Molecular methods for detecting AMR provide valuable insights; however, phenotypic resistance testing through traditional culturing remains a cornerstone of clinical practice. Culturing enables direct assessment of bacterial responses to antibiotics, which molecular techniques may overlook, particularly when resistance mechanisms are complex or not fully understood. Additionally, culturing is widely accessible and cost-effective in many clinical settings. Nonetheless, in high-risk patients with severe infections, especially when multiple risk factors for MDR strains are present, molecular-based testing may offer additional clinical value (46).

Despite the strengths of our study, including a large cohort and detailed variable analysis—limitations exist, such as its retrospective design and the possibility of unmeasured factors influencing the outcomes. Future prospective studies are necessary to confirm these risk factors and better understand their role in the development of MDR bacteria.

## Conclusion

This study identified several key risk factors for the development of MDR bacteria in head and neck infections, including female sex, oral cancer, cardiovascular disease, smoking, and insulin-treated diabetes, alongside well-established factors such as older age, immunosuppression, and prior antibiotic therapy. The prevalence of MDR bacteria was highest in coagulase-negative staphylococci, beta-hemolytic streptococci, *S. aureus*, and *Escherichia* spp. While multiple risk factors contribute to the emergence of MDR bacteria in head and neck infections, it remains uncertain whether these factors primarily drive the development of new resistant strains or simply promote colonization by existing MDR bacteria. Regardless of whether these risk factors contribute to the development of new resistant strains or the colonization by existing ones, the key clinical implication is to limit antibiotic pressure through stricter antimicrobial stewardship and targeted therapy, in order to reduce the selection and amplification of resistance mechanisms, while also supporting prevention through improved hygiene practices. Continued research is essential to better understand and effectively combat the rising threat of multidrug resistance in healthcare settings.

## Data Availability

The raw data supporting the conclusions of this article will be made available by the authors, without undue reservation.
