# Supplementary material for: Risk factors for the emergence of multidrug-resistant bacteria in patients with head and neck infections: a retrospective analysis
Source: Front Oral Health. 2026 May 22;7:1798339. doi: 10.3389/froh.2026.1798339 (PMC13236863; doi:10.3389/froh.2026.1798339)
Supplement: Supplementary file 1 [file Datasheet1.pdf]

Supplementary Material

Table 1. Distribution of bacterial isolates across resistance levels (0–3) for all identified species

Resistance category	Number of samples
0	2862 (63.71%)
1	1161 (25.85%)
2	261 (5.81%)
3	208 (4.63%)

Fig. 1. Percentages of bacterial species among MDR strains across the primary diagnoses

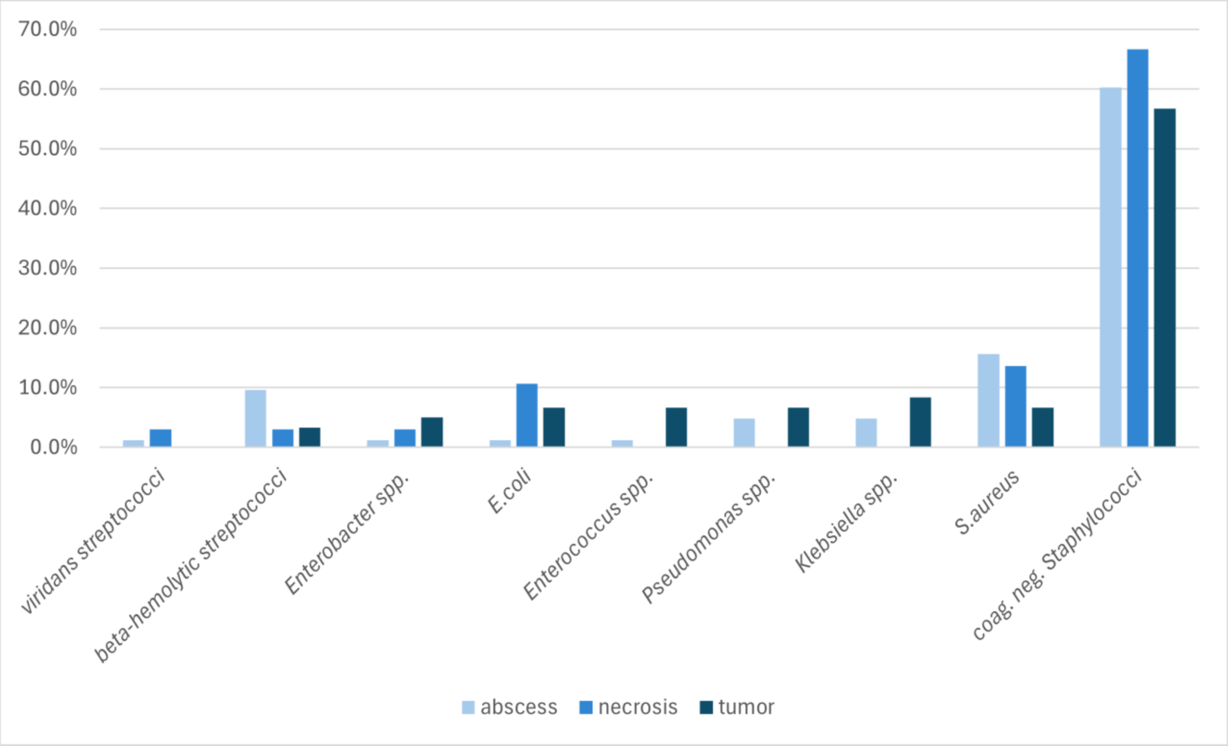

Fig. 2. Distribution of coagulase-negative staphylococcal isolates across the primary diagnoses

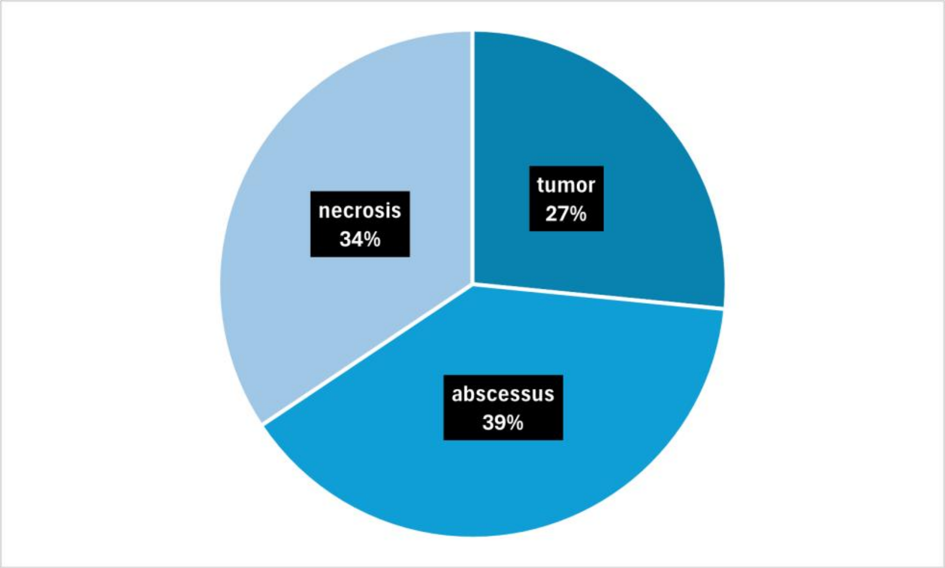

Fig.3. Proportions of various variables across resistance levels (0–3), including those identified as significant in the model. a, Prior antibiotic exposure. **b,** Diabetes with or without insulin therapy. **c,** Cardiovascular disease. **d,** Smoking (active smokers only). **e,** Primary diagnoses. **f,** Immunosuppression.

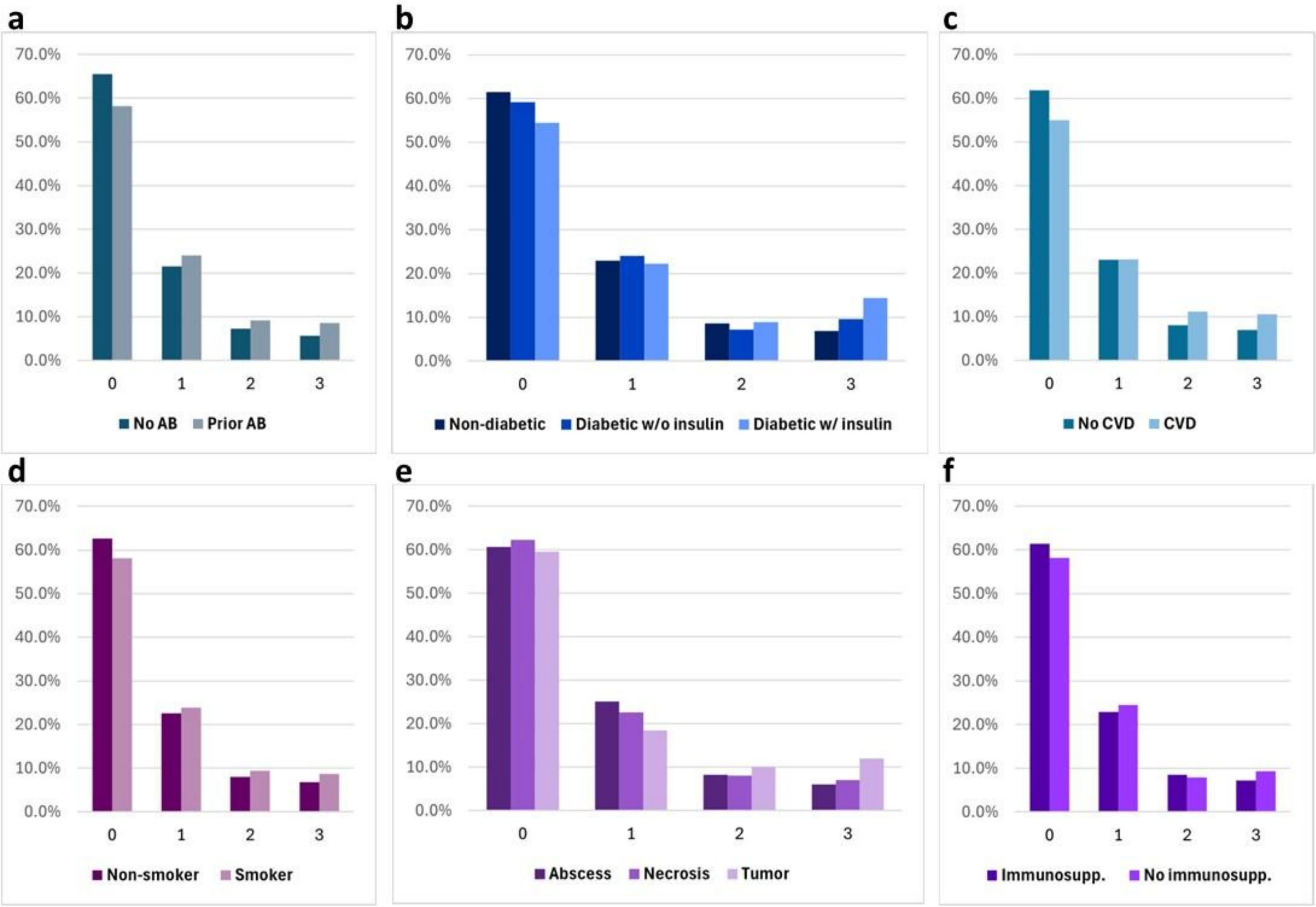

Table 2. Antimicrobial resistance rates of clinically relevant isolates according to EUCAST guidelines.

Antibiotic abbreviations: A – ampicillin, AM – amikacin, AMC – amoxicillin/clavulanic acid, AMX – amoxicillin, AZT – azithromycin, Cefaz – cefazoline, Cefep – cefepime, Cefix – cefixime, Cefot – cefotaxime, Ceftaz – ceftazidime, Ceftri – ceftriaxone, Cefur – cefuroxime, CIP – ciprofloxacin, CL – clindamycin, CLA – clarithromycin, DO – doxycycline, GE – gentamicin, GEH – high-level gentamicin, IMP – imipenem, LEV – levofloxacin, LIN – linezolid, ME – metronidazole, MEM – meropenem, OX – oxacillin, P – penicillin, RIF – Rifampicin, SXT – trimethoprim/sulfamethoxazole, TEC – teicoplanin, TIG – tigecycline, TO – tobramycin, TZP – piperacillin/tazobactam, VA – vancomycin

Less frequent Enterobacterales (e.g. *Citrobacter, Morganella, Proteus, and Serratia* spp.) were grouped for analysis purposes. Resistance patterns for *Enterococcus faecalis* and *Enterococcus faecium* were reported separately in this table due to significant and clinically relevant differences in antimicrobial susceptibility profiles.

*not reported for specific organism groups due to intrinsic resistance, lack of clinical relevance or non-routine testing in accordance with standard laboratory practice.

– : no data available, as susceptibility testing was not performed or reported by the laboratory, which applies EUCAST-based routine panels that include only selected, clinically relevant antibiotics rather than all agents within a class

		A	AM	AMC	AMX	AZT	Cefaz	Cefep	Cefix	Cefot	Ceftaz	Ceftri	Cefur	CIP	CL	CLA	DO	GE	GEH	IMP	LEV	LIN	ME	MEM	OX	P	RIF	SXT	TEC	TIG	TO	TZP	VA
***Actinomyces* spp.**		-	-	0.0%	-	-	-	-	-	-	-	-	-	-	22.0%	-	-	-	-	0.0%	-	-	*	0.0%	-	1.1%	-	-	-	-	-	0.0%	-
***Enterobacter* spp.**		-	11.7%	*	*	-	-	1.1%	-	8.9%	8.9%	8.9%	*	4.1%	-	-	-	1.4%	-	0.5%	4.1%	-	-	0.3%	-	-	-	5.1%	-	-	11.7%	9.5%	-
***Enterococcus* spp.**	*E. faecalis*	-	-	0.0%	0.0%	-	-	-	-	-	-	-	-	-	-	-	-	-	19.4%	0.0%	-	0.0%	-	-	-	-	-	-	0.0%	0.0%	-	0.0%	1.0%
	E. faecium	-	-	*	*	-	-	-	-	-	-	-	-	-	-	-	-		55.6%	*	-	0.0%	-	-	-	-	-	-	22.2%	0.0%	-	*	44.4%
***Escherichia* spp.**		-	3.9%	24.3%	56.3%	-	-	1.9%	-	6.8%	6.8%	6.8%	-	13.6%	-	-	-	2.9%	-	0.0%	13.6%	-	-	0.0%	-	-	-	21.4%	-	-	3.9%	20.4%	-
***Fusobacterium* spp.**		-	-	0.2%	-	-	-	-	-	-	-	-	-	-	22.4%	-	-	-	-	0.0%	-	-	0.6%	0.0%	-	2.0%	-	-	-	-	-	0.0%	-
***Haemophilus* spp.**		8.7%	-	0.0%	-	-	-	0.0%	0.0%	0.0%	-	0.0%	-	-	-	-	14.5%	-	-	-	13.0%	-	-	0.0%	-	-	-	5.8%	-	-	-	0.0%	-
***Klebsiella* spp.**		-	3.6%	10.0%	*	-	-	3.2%	-	5.0%	5.0%	5.0%	-	5.0%	-	-	-	2.3%	-	0.0%	5.0%	-	-	0.0%	-	-	-	5.5%	-	-	4.1%	9.5%	-
***Prevotella* spp.**		-	-	2.0%	-	-	-	-	-	-	-	-	-	-	39.9%	-	-	-	-	0.0%	-	-	0.2%	0.0%	-	6.9%	-	-	-	-	-	0.0%	-
***Pseudomonas* spp.**		-	1.9%	-	-	-	-	8.3%	-	-	9.3%	-	-	9.3%	-	-	-	0.9%	-	12.0%	17.6%	-	-	14.8%	-	-	-	-	-	-	1.9%	11.1%	-
Staphylococcus aureus		-	1.9%	13.0%	-	24.5%	13.4%	-	-	-	-	-	13.0%	11.1%	23.1%	24.5%	7.9%	0.5%	-	-	11.6%	0.0%	-	-	13.4%	-	0.5%	0.5%	-	0.5%	1.9%	-	-
**CoNS ("other" staphylococci)**		-	19.0%	28.5%	-	44.9%	28.5%	-	-	-	-	-	28.3%	20.5%	34.4%	44.9%	16.1%	12.2%	-	-	20.5%	0.0%	-	-	28.5%	-	0.7%	12.9%	1.7%	0.0%	19.2%	-	-
**Beta-hemolytic streptococci**		0.0%	-	0.0%	0.0%	26.0%	-	0.0%	-	0.0%	-	0.0%	-	-	30.0%	26.0%	62.0%	-	-	0.0%	4.0%	-	-	0.0%	-	0.0%	-	0.0%	-	-	-	0.0%	-
**Viridans streptococci ("other" streptococci)**		3.5%	-	3.6%	3.5%	*	-	0.2%	-	0.8%	-	0.8%	0.4%	-	36.2%	*	-	-	-	0.0%	-	-	-	0.0%	-	4.9%	-	-	-	-	-	2.3%	-
***Veillonella* spp.**		-	-	0.0%	-	-	-	-	-	-	-	-	-	-	24.6%	-	-	-	-	0.0%	-	-	0.6%	0.0%	-	5.2%	-	-	-	-	-	0.6%	-

Fig. 4. Pairwise comparisons of aerobic bacterial species based on resistance levels to identify those significantly associated with multidrug resistance (level 3). Statistical significance was assessed using post hoc tests with Tukey correction for multiple comparisons. STREPTOCOCCUS A= viridans streptococci ("other"), STREPTOCOCCUS B= beta-hemolytic streptococci, STAPHYLOCOCCUS A= S.aureus, STAPYLOCOCCUS E=coagulase-negative staphylococci ("other")

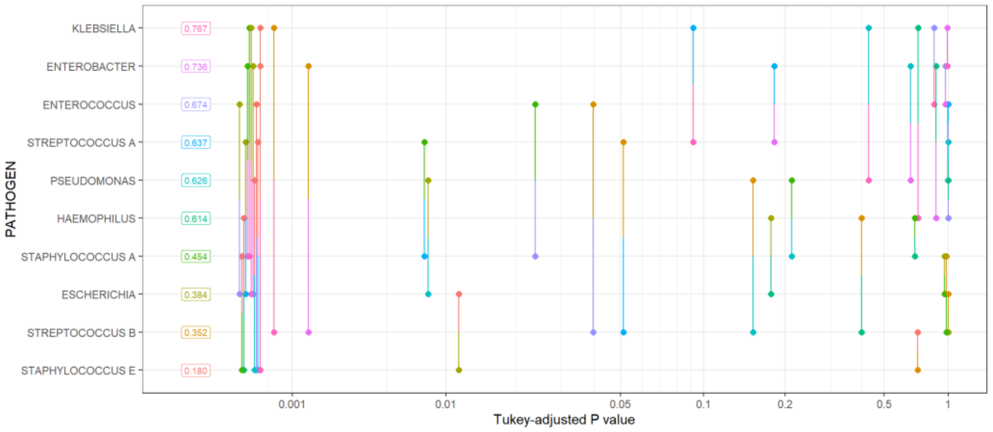

Fig. 5. Pairwise comparisons of anaerobic bacterial species based on resistance levels to identify those significantly associated with multidrug resistance (level 3). Statistical significance was assessed using post hoc tests with Tukey correction for multiple comparisons.

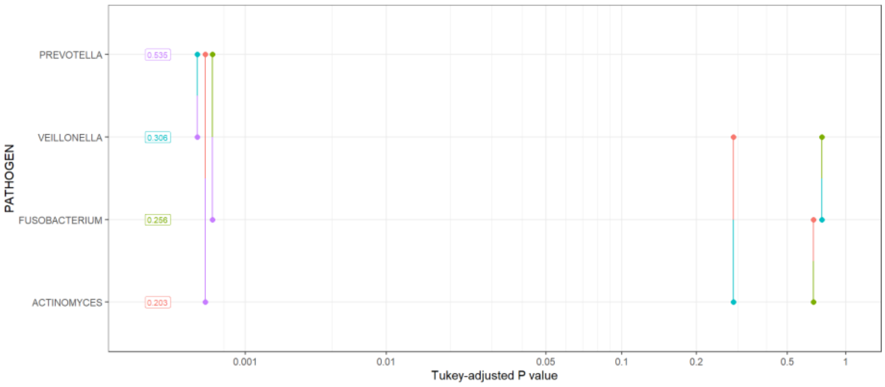

Table 3. Subgroup analysis of chemotherapy and radiotherapy as potential risk factors for MDR bacterial infections, performed in a separate model including only patients with malignancy.

**Variable**	**OR**	**95% CI**	**p-value**
SEX			
male	—	—	
female	1.28	1.02—1.60	**0.035**
HOSPITALIZATION	0.98	0.75—1.29	0.90
RECURRENCE	1.15	0.92—1.43	0.22
FOREIGN MATERIAL	0.95	0.69—1.30	0.74
PRIOR ANTIBIOTICS	1.39	1.12—1.72	**0.003**
RENAL FAILURE	1.09	0.56—2.12	0.80
POOR ORAL HYGIENE	0.90	0.67—1.22	0.50
DIABETES			
non-diabetic	—	—	
diabetic without insulin	1.05	0.78—1.41	0.74
diabetic with insulin	1.48	0.87—2.50	0.15
IMMUNOSUPPRESSION	1.43	1.09—1.89	**0.010**
SMOKING	1.21	0.94—1.55	0.14
ALCOHOL	0.73	0.51—1.05	0.093
HIGH CRP	1.78	0.65—4.87	0.26
CARDIOVASCULAR DISEASE	1.24	0.93—1.64	0.14
ANTIRESORPTIVE THERAPY	0.74	0.58—0.96	**0.024**
CHEMOTHERAPY	0.79	0.62—1.01	0.065
RADIOTHERAPY	1.08	0.85—1.36	0.53
AGE	1.00	0.99—1.02	0.38

Table 4. Separate model including only patients in the necrosis group

Variable	OR	95% CI	p-value
SEX			
male (ref)	—	—	
female	1.21	0.92–1.58	0.17
HOSPITALIZATION	0.99	0.74–1.32	0.93
RECURRENCE	1.17	0.90–1.53	0.25
FOREIGN MATERIAL	1.27	0.85–1.88	0.24
PRIOR ANTIBIOTICS	1.35	1.04–1.75	**0.025**
PRIOR MALIGNANCY	0.85	0.57–1.26	0.41
RENAL FAILURE	1.41	0.63–3.18	0.40
POOR ORAL HYGIENE	1.19	0.80–1.76	0.39
DIABETES			
non-diabetic (ref)	—	—	
diabetic without insulin	1.03	0.70–1.51	0.89
diabetic with insulin	1.38	0.71–2.65	0.34
IMMUNOSUPPRESSION	1.37	1.03–1.84	**0.031**
SMOKING	1.23	0.91–1.66	0.18
ALCOHOL	1.08	0.65–1.78	0.77
HIGH CRP	1.61	0.36–7.22	0.53
CARDIOVASCULAR DISEASE	1.32	0.92–1.91	0.14
ANTIRESORPTIVE THERAPY	0.78	0.58–1.06	0.11
AGE	1.01	0.99–1.02	0.35

Table 5. Separate model including only patients in the abscess group

Variable	OR	95% CI	p-value
SEX			
male (ref)	—	—	
female	1.26	1.01, 1.58	**0.044**
HOSPITALIZATION	1.09	0.85, 1.39	0.50
RECURRENCE	1.07	0.80, 1.43	0.66
FOREIGN MATERIAL	1.76	1.16, 2.67	**0.008**
PRIOR ANTIBIOTICS	1.61	1.27, 2.04	**<0.001**
PRIOR MALIGNANCY	1.51	0.94, 2.40	0.087
RENAL FAILURE	0.59	0.15, 2.31	0.45
POOR ORAL HYGIENE	1.30	0.95, 1.76	0.10
DIABETES			
non-diabetic (ref)	—	—	
diabetic without insulin	0.86	0.57, 1.31	0.49
diabetic with insulin	1.74	0.94, 3.21	0.078
IMMUNOSUPPRESSION	1.59	0.90, 2.83	0.11
SMOKING	1.23	0.96, 1.58	0.10
ALCOHOL	0.86	0.55, 1.35	0.51
HIGH CRP	1.17	0.80, 1.71	0.40
CARDIOVASCULAR DISEASE	1.05	0.68, 1.62	0.82
ANTIRESORPTIVE THERAPY	0.86	0.35, 2.12	0.74
INCISION TYPE			
extraoral	—	—	
intraoral	0.99	0.77, 1.26	0.91
AGE	1.01	1.00, 1.01	**0.049**

Table 6. Analysis of risk factors for AMR among anaerobic bacterial isolates.

Variables	Odds Ratios	CI	p value
PRIMARY DIAGNOSIS [TUMOR]	0.67	0.26 – 1.72	0.404
SEX [female]	1.46	0.95 – 2.25	0.083
HOSPITALIZATION	1.10	0.69 – 1.75	0.687
RECURRENCE	1.01	0.61 – 1.66	0.980
FOREIGN BODY	1.70	0.83 – 3.49	0.145
PRIOR AB	2.35	1.60 – 3.44	**<0.001**
PRIOR MALIGNANCY	1.03	0.46 – 2.29	0.940
RENAL FAILURE	6.54	1.46 – 29.41	**0.014**
POOR ORAL HYGIENE	1.29	0.73 – 2.28	0.375
DIABETES [diabetic without insulin]	1.02	0.50 – 2.10	0.950
DIABETES [diabetic with insulin]	0.57	0.19 – 1.73	0.319
IMMUNOSUPPRESSION	1.02	0.53 – 1.96	0.963
SMOKING	0.81	0.51 – 1.28	0.372
ALCOHOL	1.22	0.53 – 2.78	0.638
HIGH CRP	0.89	0.35 – 2.25	0.800
CARDIOVASCULAR DISEASE	0.92	0.46 – 1.82	0.804
ANTIRESORPTIVE THERAPY	1.32	0.63 – 2.75	0.456
AGE	0.99	0.98 – 1.00	0.170
